# Resource use and outcome in critically ill patients with hematological malignancy: a retrospective cohort study

**DOI:** 10.1186/cc6921

**Published:** 2008-06-06

**Authors:** Tobias M Merz, Pascale Schär, Michael Bühlmann, Jukka Takala, Hans U Rothen

**Affiliations:** 1 Department of Intensive Care Medicine, Royal North Shore Hospital of Sydney, University of Sydney, St Leonards, 2065 NSW, Australia; 2 Department of Internal Medicine, Inselspital, Bern University Hospital and University of Bern, 3010 Bern, Switzerland; 3 Department of Medical Oncology, Inselspital, Bern University Hospital and University of Bern, 3010 Bern, Switzerland; 4 Department of Intensive Care Medicine, Inselspital, Bern University Hospital and University of Bern, 3010 Bern, Switzerland

## Abstract

**Introduction:**

The paucity of data on resource use in critically ill patients with hematological malignancy and on these patients' perceived poor outcome can lead to uncertainty over the extent to which intensive care treatment is appropriate. The aim of the present study was to assess the amount of intensive care resources needed for, and the effect of treatment of, hemato-oncological patients in the intensive care unit (ICU) in comparison with a nononcological patient population with a similar degree of organ dysfunction.

**Methods:**

A retrospective cohort study of 101 ICU admissions of 84 consecutive hemato-oncological patients and 3,808 ICU admissions of 3,478 nononcological patients over a period of 4 years was performed.

**Results:**

As assessed by Therapeutic Intervention Scoring System points, resource use was higher in hemato-oncological patients than in nononcological patients (median (interquartile range), 214 (102 to 642) versus 95 (54 to 224), *P *< 0.0001). Severity of disease at ICU admission was a less important predictor of ICU resource use than necessity for specific treatment modalities. Hemato-oncological patients and nononcological patients with similar admission Simplified Acute Physiology Score scores had the same ICU mortality. In hemato-oncological patients, improvement of organ function within the first 48 hours of the ICU stay was the best predictor of 28-day survival.

**Conclusion:**

The presence of a hemato-oncological disease *per se *is associated with higher ICU resource use, but not with increased mortality. If withdrawal of treatment is considered, this decision should not be based on admission parameters but rather on the evolutional changes in organ dysfunctions.

## Introduction

Patients with hematological malignancy who are admitted to the intensive care unit (ICU) due to complications of the underlying malignant disease often have a prolonged stay in the ICU [1] and are believed to have a less favorable prognosis [2] than nononcological patients. In general adult ICU populations, prolonged stay has been reported to be associated with a disproportionate use of resources [3]. Information on resource use of hemato-oncological patients requiring intensive care is relatively scarce [4,5], however, and comparisons with other nononcological intensive care patient groups do not exist. The paucity of data on resource use in hemato-oncological patients and the perceived poor outcome can lead to uncertainty over the extent to which intensive care treatment is appropriate in this patient group [6-8]. Decisions to admit cancer patients to the ICU are exceptionally complex, as the chances of potentially curative cancer therapy or long-term palliation must be weighed against the associated risk of very high morbidity or mortality and thus possible futile use of more and more limited resources.

Reported ICU mortality rates of critically ill hemato-oncological patients vary widely, from 10% up to 50% depending on the studied population [9-11]. The prognostic value of various clinical indicators – such as age, primary disease, chronic health status, cardiovascular failure, renal insufficiency, leucopenia or recent bacteremia [12] – is in dispute. Likewise, the value of various scoring systems applied at the time of ICU admission to predict outcome is controversial [13,14]. Extending any prediction to individual patients remains a clinical decision for which specific outcome indicators provide little help.

Furthermore, treatments and outcomes of various malignancies have changed, suggesting that re-evaluation of indications and outcomes of intensive care for this patient group is necessary. Although a multicenter approach is considered necessary to generate the number of patients needed to evaluate any prognostic indicator, a more detailed evaluation of resource utilization may benefit from a single-center analysis, which avoids the effect of variability between different ICUs [15].

Accordingly, the primary aim of the present single-center study was to assess the amount of resources used per patient for hemato-oncological and nononcological emergency admissions to the ICU. A secondary aim was to explore the survival of hemato-oncological patients depending on their pre-existing comorbidities and on the severity of acute illness on admission and during the course of the ICU stay, and in comparison with a nononcological patient population with a similar degree of organ dysfunction.

## Materials and methods

### Setting

The Bern University Hospital, Switzerland, is a 960-bed tertiary care referral hospital. The Department of Intensive Care Medicine is the sole provider of intensive care for adult patients in the hospital. The department comprises 30 beds, and is operated as a closed unit. Care is offered for all types of surgical, trauma and medical patients, except major burn injuries. Admission of hemato-oncological patients to the ICU takes place after consultation with the treating oncologist and a senior intensive care physician. Criteria for admission are largely identical for hemato-oncological patients and for nononcological patients [16]. We tend to abstain from admission of a hemato-oncological patient in the case of progressed malignancy and short expected survival time (<3 months). The Department of Medical Oncology includes an inpatient section with 50 beds and an outpatient section handling approximately 13,000 consultations per year.

### Patients and data collection

The collective of hemato-oncological patients included in the present retrospective cohort study consisted of all patients with a primary diagnosis of leukemia, lymphoma or myeloma admitted to the ICU from July 2001 to July 2005 due to a severe deterioration in their general condition. Patients referred to the ICU solely for rhythm monitoring during a chemotherapeutical intervention were excluded. The collective of nononcological patients evaluated for comparison consisted of all medical patients admitted as emergencies to the ICU during the same period of time. Patients admitted after elective or emergency surgery were excluded from the analysis. For comparison of hemato-oncological and nononcological patient survival and resource use (see below), readmissions occurring within 48 hours of discharge from the ICU were considered with the initial admission, whereas readmissions beyond 48 hours were analyzed as new cases [17].

The ICU stay parameters for all patients were collected from the ICU database. These parameters included age, sex, date of admission to the hospital, date of each ICU admission throughout the hospital stay, reason for ICU admission, date of and status at ICU discharge, Simplified Acute Physiology Score (SAPS II) [18] calculated for the first 24 hours of the ICU stay, and the amount of Therapeutic Intervention Scoring System (TISS-28) points [19] accumulated throughout the ICU stay. As treatment intensity often changes markedly, even within 1 day, we calculated the TISS-28 score once per nursing shift (that is, every 8 hours) [3,20]. Patient-related direct costs were calculated based on the hospital cost accounting, and amounted to 38 Swiss Francs per TISS-28 point.

The Sequential Organ Failure Assessment (SOFA) score [21] for each day of the ICU stay was collected from information in the medical records and was available only for hemato-oncological patients, as it is not part of the ICU database. To assess the change during the first 48 hours of the ICU stay, the difference between the patients' SOFA score at ICU admission and after the first 24 and 48 hours of the ICU stay was calculated. Stabilization of the patients' condition was defined as an unchanged or decreased SOFA score, and deterioration was defined as an increased SOFA score. The use of renal replacement therapy (intermittent hemodialysis or continuous hemodiafiltration) and mechanical ventilation were also recorded.

Additional data on hemato-oncological patients, collected from their medical records, included primary oncological diagnosis, presence of neutropenia (defined as minimal absolute neutrophil count <500/μl) and the type of anticancer treatment. The type of hematological malignancy was categorized into high-grade malignancy (acute myelogenous leukemia, acute lymphoblastic leukemia and high-grade non-Hodgkin's lymphoma) and low-grade malignancy (all other types of hematologic malignancies) [12].

To evaluate underlying comorbidities we used the Adult Comorbidity Evaluation-27 system [22], which contains 12 comorbid ailments (cardiovascular, respiratory, gastrointestinal, renal, endocrine and neurological systems, psychiatric, rheumatological and immunological disorders, malignancy, substance abuse, and body weight). Each of these comorbidities was classified by Grade 0 to 3 (0 = no comorbidity, 1 = mild, 2 = moderate, 3 = severe comorbidity). To evaluate the overall comorbidity index we ranked the highest single ailment, except when two or more grade 2 ailments occurred in different organ systems. In this case we designated the overall comorbidity score as grade 3. Survival at 28 days and 1 year after ICU admission was obtained from the patient files of the oncological outpatient section.

### Statistical analysis

Data are presented as the mean ± standard deviation or the median (interquartile range) as appropriate. Outcome groups on the basis of hospital survival/nonsurvival and resource use in nononcological patients and hemato-oncological patients were compared using an unpaired *t *test and the Mann–Whitney test for continuous variables with normal and skewed distributions, respectively. Fisher's exact test or the Pearson chi-square test was used for categorical variables. The correlation between reason for ICU admission, Adult Comorbidity Evaluation-27 score as well as type of anticancer treatment and outcome was assessed by applying categorical logistic regression.

To compare resource use in nononcological patients and in hemato-oncological patients after correction for severity of organ dysfunction at ICU admission, a stepwise multiple linear regression model was applied including SAPS II and the presence or absence of hematological malignancy as predictors.

To compare ICU mortality in nononcological patients and in hemato-oncological ICU patients, a stepwise multiple logistic regression model was applied, including SAPS II and the presence or absence of hematological malignancy as predictors. To avoid colinearity, for all regression analyses including nononcological patients, SAPS II values for hemato-oncological patients were reduced by 10 points to balance the number of SAPS II points added in the original score for the presence of hematological malignancy. For the direct comparison of the prognostic value of SOFA scores and SAPS II and the change of the SOFA score in the first 24 and 48 hours, Pearson's chi-square test was used. The same test was used to analyze the effect of use of mechanical ventilation and renal replacement therapy with respect to 28-day mortality. All these results are reported as contingency coefficients [23]. Accordingly, the continuous variables SOFA score and SAPS II had to be dichotomized. This was achieved using receiver operating characteristic curves, plotting sensitivity versus 1 – specificity. Based on these receiver operating characteristic curves, we defined cutoff values for SAPS II and the SOFA scores that discriminated best between survivors and nonsurvivors at day 28.

Additionally, the independent predictive value of the change in SOFA score and of absolute organ failure scores at ICU admission was assessed in two logistic regression models. The first model analyzed the change in SOFA score and admission SAPS II value; the second model included the change in the SOFA score and the absolute admission SOFA score.

In all analyses, *P *< 0.05 was considered statistically significant. Statistical analyses were performed using the software package SPSS, version 13.0 (SPSS, Inc., Chicago, IL, USA).

### Patient consent

The study was approved by the Ethical Committee of the Bern University Hospital and adhered to the tenets of the Declaration of Helsinki. All patients of the Bern University Hospital are informed on admission that they can specify whether data related to their stay can be used in retrospective studies; data of patients who declined were not included in the study.

## Results

### Baseline characteristics of nononcological patients

During the study period, 10,628 nononcological patients were admitted to the ICU, accounting for a total of 12,065 admissions. After exclusion of patients admitted after elective or emergency surgery, a total of 3,808 medical emergency ICU admissions of 3,478 patients were included in the further analysis. Table 1 presents the characteristics of these nononcological patient admissions, stratified into survivors and nonsurvivors of the ICU stay. As expected, nonsurviving patients had higher SAPS II at ICU admission and higher rates of mechanical ventilation and renal replacement therapy, but the length of ICU stay was very similar in the two patient groups. The 1,542 nononcological patients had a stay of less than 24 hours, and 181 (11.7%) of these patients died in the ICU.

**Table 1:** Characteristics of nononcological emergency intensive care unit (ICU) admissions

Characteristic	Admissions of ICU survivors (n = 3402)	Admissions of ICU nonsurvivors (n = 406)	*P *value
Age (years)	56.9 ± 17.6	61.2 ± 16.7	0.14
Male/female (%)	66.5/33.5	67.3/32.7	0.78
Reason for ICU admission (%)			<0.0001
Sepsis	11.6	21.7	
Respiratory failure	9.5	5.9	
Cardiovascular failure	35.5	34.6	
Neurological failure	20.5	21.2	
Abdominal event	10.3	8.3	
Other	12.5	8.3	
SAPS II at ICU admission	29 (20 to 41)	63 (51 to 77)	<0.0001
Mechanically ventilated patients (%)	46.6	93.1	<0.0001
Renal replacement therapy (%)	6.9	15.8	<0.0001
ICU length of stay (days)	1.1 (0.7 to 2.6)	1.1 (0.5 to 3.1)	0.019

Data expressed as the percentage, the mean ± standard deviation or the median (interquartile range). SAPS II, Simplified Acute Physiology Score. *P*, significance value for comparison of hospital survivors and nonsurvivors.

### Baseline characteristics of hemato-oncological patients

During the study period, 1,415 oncological patients were admitted to the Department of Medical Oncology, accounting for a total of 2,416 admissions. Eighty-four of these patients (5.9%), meeting the study entry criteria of primary diagnosis of hematological malignancy (leukemia, lymphoma or myeloma), had to be admitted to the ICU due to acute deterioration and were included in the study. Seventy patients were admitted to intensive care once, 14 patients were admitted twice, and one patient was admitted three times. The resulting total of 101 hemato-oncological patient ICU admissions accounted for 2.6% of all medical emergency admissions to the ICU. Six hemato-oncological patients had an ICU length of stay of less than 24 hours, and three of these patients (50%) died in the ICU.

Table 2 presents the characteristics of hemato-oncological patients, stratified as survivors and nonsurvivors of the ICU stay. Nonsurvivors had a higher SAPS II at ICU admission and a higher rate of mechanical ventilation and renal replacement therapy. There was no significant difference between survivors and nonsurvivors with respect to age, preexisting comorbidities, distribution of primary diagnosis and occurrence of neutropenia.

**Table 2:** Characteristics of emergency intensive care unit (ICU) admissions of patients with hematologic malignancy

	Admissions of ICU survivors (n = 78)	Admissions of ICU nonsurvivors (n = 23)	*P *value
Age (years)	47.6 ± 15.6	53.7 ± 9.6	0.84
Male/female (%)	59.0/41.0	65.2/34.8	0.59
Reason for ICU admission (%)			0.15
Sepsis	47.4	56.5	
Respiratory failure	24.4	30.4	
Cardiovascular failure	9.0	0	
Neurological failure	1.3	13.0	
Abdominal event	6.4	0	
Other	11.5	0	
Type of hematological malignancy			0.51
Acute myelogenous leukemia	39 (50%)	16 (69.6%)	
Acute lymphoblastic leukemia	11 (14.1%)	0	
Chronic myelogenous leukemia	2 (2.6%)	0	
Non-Hodgkin's lymphoma/myeloma	23 (29.5%)	6 (26.1%)	
Hodgkin's lymphoma	3 (3.9%)	0	
Myelodysplastic syndrome	0	1 (4.4%)	
Grading of hematological malignancy			1.0
High grade	60 (77%)	18 (78%)	
Low grade	18 (33%)	5 (22%)	
Type of treatment (before ICU admission)*a*			0.41
Chemotherapy	72 (92.3%)	20 (87.0%)	
Radiation	9 (11.5%)	0	
Surgical procedures	4 (5.1%)	1 (4.3%)	
Autologous/allogeneic stem cell transplantation	9 (11.5%)	3 (13.0%)	
Disease stage			0.87
Initial diagnosis	57 (73.1%)	18 (78.3%)	
More advanced disease/relapse	15 (19.2%)	3 (13.0%)	
Complete remission	1 (1.3%)	0	
Chronic disease	4 (5.1%)	2 (8.7%)	
Other/not applicable	1 (1.3%)	0	
Rate of neutropenia	59.0%	60.9%	1.00
ACE-27 0/1/2/3 points (%)	53.9/21.8/17.9/6.4	43.5/21.7/26.1/8.7	0.32
ACE-27 0 to 1	59 (76%)	15 (65%)	0.42
ACE-27 2 to 3	19 (24%)	8 (35%)	
SAPS II at ICU admission	46 (34 to 56)	76 (63 to 90)	<0.0001
Mechanical ventilation (%)	43.5	95.6	<0.0001
Renal replacement therapy (%)	3.8	39.1	<0.0001
ICU length of stay (days)	2 (1 to 6)	6 (2 to 11)	0.02

Data expressed as percentage, number (percentage), mean ± standard deviationor median (interquartile range). ACE-27, Adult Comorbidity Evaluation-27; SAPS II, Simplified Acute Physiology Score. *P*, significance value for comparison of hospital survivors and nonsurvivors. *a*Some patients received treatments in combination.

### Comparison of resource use in hemato-oncological patients and in nononcological patients

Table 3 presents resource use measured by the total TISS-28 points in hemato-oncological patients and in nononcological patients. The total resource use varied considerably from patient to patient (coefficient of variation = 144% for hemato-oncological patients, coefficient of variation = 191% for medical patients). Hemato-oncological patients consumed significantly more ICU resources than nononcological patients. This difference was generated by the hemato-oncological patients' longer ICU stays and higher nursing intensity per nursing shift. Owing to the higher resource use per patient, combined with a higher mortality, the number of TISS-28 points per surviving patient was 2.4 times higher in hemato-oncological patients than in nononcological patients. Average total direct costs per ICU admission were 10,070 Swiss Francs in nononcological patients and 24,206 Swiss Francs in hemato-oncological patients.

**Table 3:** Evaluation of resource use in hemato-oncological and nononcological patients admitted to the intensive care unit

	Admitted hemato-oncological patients	Admitted nononcological patients	*P *value
Number of admissions	101	3,808	
Total TISS-28 points	214 (102 to 642)	95 (54 to 224)	<0.0001
TISS-28 points per nursing shift	28 (22 to 32)	23 (18 to 28)	<0.0001
Intensive care unit length of stay (days)	2 (1 to 6)	1.1 (0.7 to 2.6)	<0.0001
TISS-28 points per surviving patient	637	265	

Data expressed as the median (interquartile range). TISS-28, Therapeutic Intervention Scoring System.

In a stepwise multiple linear regression model, SAPS II (*b *= 5.75, β = 0.238, *P *< 0.0001) and the presence of hemato-oncological disease (*b *= 212.3, β = 0.072, *P *< 0.0001) were significant predictors of resource use. The variation in SAPS II, however, only accounted for 5.9% (*R*^2 ^= 0.059, *P *< 0.0001) of the variation in total TISS-28 points. After inclusion of hemato-oncological disease as a predictor, the model accounted for 6.4% of overall variation in resource use (*R*^2 ^= 0.064, *P *< 0.0001).

### Organ failure, treatment modalities and resource use in hemato-oncological patients

Hemato-oncological patients had a higher SAPS II score at ICU admission than nononcological patients (48 (36 to 65) versus 31 (21 to 45), *P *< 0.0001). The rates of mechanical ventilation (54.4% versus 51.5%; *P *= 0.44) and renal replacement therapy (11.9% versus 7.9%, *P *= 0.21) were similar to those of nononcological patients. Table 4 presents the correlations of ICU treatment modalities and severity of organ failure at admission and during the course of the ICU stay to the total ICU resource use in hemato-oncological patients. All parameters defined by the severity of organ failure, except occurrence of neutropenia, show a similar significant correlation to total ICU resource use. Necessity for renal replacement therapy showed a moderate association to total ICU resource use, whereas mechanical ventilation was associated with the highest increase of resource use of all evaluated parameters. Higher ICU resource use, as measured by TISS-28 points and longer ICU length of stay, was not associated with 28-day survival after correction for severity of disease measured by SAPS II.

**Table 4:** Correlation of intensive care unit (ICU) treatment modalities and severity of organ failure to resource use in hemato-oncological patients

	Pearson's chi-square test	Odds ratio (95% confidence interval)	Contingency coefficient	*P *value
Renal replacement therapy during ICU stay (yes/no)	6.23	6.13 (1.27 to 29.59)	0.25	0.015
Mechanical ventilation during ICU stay (yes/no)	37.42	17.95 (6.49 to 49.67)	0.69	<0.0001
Neutropenia during ICU stay (yes/no)	0.13	1.04 (0.48 to 2.30)	0.01	0.98
ICU admission SAPS II (≤ 62/>62)	15.87	6.92 (2.50 to 19.13)	0.39	<0.0001
ICU admission SOFA score (≤ 12/>12)	14.46	6.39 (2.30 to 17.67)	0.38	<0.0001
SOFA score increased 24 hours after ICU admission (yes/no)	8.90	3.64 (1.53 to 8.66)	0.30	0.004
SOFA score increased 48 hours after ICU admission (yes/no)	15.35	6.29 (2.38 to 16.61)	0.39	<0.0001

SAPS II, Simplified Acute Physiology Score; SOFA, Sequential Organ Failure Assessment.

### Comparison of survival of hemato-oncological patients and nononcological patients

Hemato-oncological patients showed higher hospital mortality than nononcological patients (33.7% versus 10.7%, *P *< 0.0001). The prognostic significance of the severity of illness at ICU admission, as measured by SAPS II, and the presence of a hemato-oncological disease were assessed in a multiple logistic regression model. The goodness-of-fit of this model was modest (Cox and Snell *R*^2 ^= 0.21, Nagelkerke *R*^2 ^= 0.42, chi-square = 914.2, *P *< 0.0001). In this analysis only SAPS II (odds ratio = 1.086, 95% confidence interval (CI) = 1.079 to 1.094, *P *< 0.0001) was a significant predictor of hospital mortality. The presence of a hemato-oncological disease was not associated with an additional risk of adverse outcome (odds ratio = 0.59, 95% CI = 0.32 to 1.08, *P *= 0.09).

### Predictors of survival in hemato-oncological patients

The overall 28-day survival rate in hemato-oncological patients after ICU admission was 70.2%. The hospital survival rate was 66.4% and the 90-day survival rate was 60.3%. Both SAPS II (area under the curve = 0.799, 95% CI = 0.702 to 0.896, *P *< 0.0001) and the SOFA score (AUC = 0.688, 95% CI = 0.571 to 0.804, *P *= 0.002) were significant predictors of 28-day mortality. The best cut-off values were identified as SAPS II = 62 (sensitivity = 0.67, 95% CI = 0.47 to 0.82; specificity = 0.86, 95% CI = 0.75 to 0.93) and SOFA score = 12 (sensitivity = 0.50, 95% CI = 0.31 to 0.69; specificity = 0.80, 95% CI = 0.70 to 0.89).

Table 5 illustrates that nearly all evaluated parameters determined by the severity of acute illness or concomitant organ failure at admission or during the course of the ICU stay show a significant correlation with the 28-day outcome. The best predictor of an adverse outcome was the ongoing deterioration of the patient's condition, expressed by an increase in the SOFA score during the first 48 hours, followed by high SAPS II. This result was confirmed by logistic regression models including the change in SOFA score during the first 48 hours of the ICU stay and the admission SAPS II or SOFA score as predictors, and including 28-day mortality as outcome parameter. The first model indicated an odds ratio of 1.039 (95% CI = 1.007 to 1.071, *P *= 0.015) for admission SAPS, and an odds ratio of 8.15 (95% CI = 2.53 to 26.20, *P *< 0.0001) for the change in SOFA score. The second model resulted in an odds ratio of 1.09 (95% CI = 0.0.90 to 1.32, *P *= 0.12) for the admission SOFA score, and an odds ratio of 13.16 (95% CI = 4.16 to 41.62, *P *< 0.0001) for the change in SOFA score.

**Table 5:** Correlation of severity of organ failure and treatment modalities to 28-day survival in hemato-oncological patients

	Pearson's chi-square test	Odds ratio (95% confidence interval)	Contingency coefficient	*P *value
Renal replacement therapy during ICU stay (yes/no)	13.38	9.71 (2.40 to 39.33)	0.36	0.001
Mechanical ventilation during ICU stay (yes/no)	10.42	4.87 (1.77 to 13.38)	0.32	0.002
Neutropenia during ICU stay (yes/no)	0.11	1.16 (0.49 to 2.77)	0.03	0.73
ICU admission SAPS II (≤ 62/>62)	27.92	12.2 (4.43 to 33.56)	0.52	<0.0001
ICU admission SOFA score (≤ 12/>12)	9.45	4.07 (1.61 to 10.26)	0.31	0.004
SOFA score increased 24 hours after ICU admission (yes/no)	8.22	3.57 (1.46 to 8.74)	0.28	0.006
SOFA score increased 48 hours after ICU admission (yes/no)	34.2	24.4 (8.77 to 67.91)	0.58	<0.0001

SAPS II, Simplified Acute Physiology Score; SOFA, Sequential Organ Failure Assessment.

## Discussion

In our institution, hemato-oncological patients consumed significantly more resources per patient than the mixed population of nononcological emergency patients admitted in the same period of time. This difference persisted even after correction for severity of illness at ICU admission, and was due to the hemato-oncological patients' longer ICU stays as well as to their higher treatment intensity. Severity of disease at ICU admission was a weak predictor of resource use in both patient groups. In hemato-oncological patients, the total ICU resource use was more dependent on specific costly treatment modalities, especially mechanical ventilation, than on severity of disease. Our findings concur with other reports in general ICU populations [24,25] and in critically ill oncological patients [8] showing that treatment complexity rather than disease severity is the most important determinant of ICU resource use.

The use of more intensive care resources and a longer ICU length of stay were not associated with an improved outcome. This might be explained by the fact that some forms of organ failure (such as respiratory failure) might lead to higher use of ICU resources than others (such as circulatory or neurologic failure), but can be associated with a similar risk of death. Similarly, a short length of stay might be associated with rapid stabilization and discharge of the patient as well as with further deterioration and death shortly after ICU admission. Intensive care was necessary in only a small proportion of our hemato-oncological patients. Although we do not have access to detailed cost estimations, we assume that intensive care costs represent only a small fraction of the overall resource use for the treatment of hemato-oncological patients in our institution.

The existence of a malignant disease *per se *was not associated with higher ICU mortality. This observation indicates that the higher grade of organ dysfunction of hemato-oncological patients compared with nononcological patients determines their higher mortality, rather than the presence of the malignant disease. The reliable prediction of the chance of survival of an individual critically ill hemato-oncological patient prior to ICU admission is difficult, and the clinical judgment of intensivists is often inaccurate [26]. Although a more severe and prolonged course of disease is significantly correlated with adverse outcome, patients with a prolonged stay in the ICU still may attain an acceptable level of health-related quality of life [20]. Critically ill hemato-oncological patients should therefore not be deprived of intensive care solely due to their underlying malignant disease or the expected high costs.

Prior studies in critically ill hemato-oncological patients have shown that more comorbidities [27] and the degree of acute organ dysfunction are important predictors of mortality, and have a higher correlation to unfavorable outcome than the characteristics of the underlying malignancy (that is, advanced age, metastatic or progressive disease, neutropenia, and bone marrow transplantation) [28-30]. In our patients we did not find a trend toward higher mortality in patients with more comorbidities and outcome was not associated with age, the primary reason for ICU admission or the type of hemato-oncological treatment associated with outcome. The severity of acute illness and acute organ dysfunction, represented by the admission SAPS II and the admission SOFA score, respectively, were also closely correlated with 28-day survival in our patients. Patients whose condition stabilized during the first 48 hours of the ICU stay, however, showed a markedly higher survival rate than patients who continued to deteriorate despite all intensive care efforts. This finding was independent of the admission SAPS II and the admission SOFA score, furthermore confirming that the course of disease in the first 48 hours after admission seems to be as important as, if not more important than, the admission parameters in determining outcome [31].

Our findings are in contrast to the results of Lamia and colleagues [32], who compared admission values and changes after 72 hours in different organ failure scores in hemato-oncological patients admitted to the ICU, and found that admission scores and changes in score perform similarly in predicting outcome. This observation can possibly be explained by the fact that these authors defined the change in severity of acute illness as a ratio of the organ failure scores on day 1 and on day 3, rather than using the absolute difference of the scores. With this method, the same absolute change results in different ratios depending on the degree of organ failure on admission, thereby diminishing the discriminative value of the score changes for higher admission scores. An improvement or normalization of organ function might have a similar or even more important impact in patients with a high degree of organ dysfunction.

The retrospective nature of the study and the relatively small patient population are limitations of the present study. The 28-day survival rate of our hemato-oncological population was higher than survival rates reported in other collectives of critically ill hemato-oncological patients [26,28,33-35]. The type and extent of the underlying malignancy [9,36,37] and the patient's age [38] influence the outcome of critically ill cancer patients; our results may therefore be applicable to hemato-oncological patients but not to patients suffering from other types of malignancies. In addition, there may have been patients who were not admitted to the ICU because of progressed malignancy and short expected survival time or a do-not-resuscitate order, and who were not included for analysis.

In terms of the strengths of our study, the single-center design allowed for the calculation of the occurrence rate of ICU admissions for the whole hemato-oncological inpatient service and for the exact evaluation of comorbidities. We were therefore able to control for acute and chronic confounding factors as well as for the presence of malignancy when comparing mortality of hemato-oncological patients and of nononcological patients.

## Conclusion

In the examined population, critically ill hemato-oncological patients had a longer ICU stay and consumed more critical care resources than nononcological patients. Resource use in the ICU depended more on the need for specific costly treatment modalities during the ICU stay than on the extent of organ failure at ICU admission; the prediction of resource use is therefore not possible at the time of admission.

After adjustment for the severity of acute disease on admission to the ICU, the presence of a hemato-oncological disease *per se *was not associated with a higher risk of ICU mortality. Further, improvement of organ function early after ICU admission, rather than the initial severity of disease, was the most important prognostic factor for outcome. Accordingly, we suggest that critically ill hemato-oncological patients should be admitted to the ICU regardless of their underlying malignancy. If withdrawal of treatment is considered in a specific patient, a decision should not be based on admission parameters but rather on the evolutional changes in organ dysfunctions.

## Key messages

• Hemato-oncological patients consume more critical care resources and have a longer ICU stay than nononcological patients.

• Improvement of organ function early after ICU admission is the most important prognostic factor for outcome in hemato-oncological patients.

• In critically ill patients, the presence of a hemato-oncological disease *per se *is not associated with a higher risk of ICU mortality.

## Abbreviations

95% CI = 95% confidence interval; ICU = intensive care unit; SAPS II = Simplified Acute Physiology Score II; SOFA = Sequential Organ Failure Assessment; TISS-28 = Therapeutic Intervention Scoring System.

## Competing interests

The authors declare that they have no competing interests.

## Authors' contributions

TMM and PS contributed equally to this work. TMM, PS, HUR and JT participated in the design of the study. PS and MB collected all data on hemato-oncological patients. TMM and PS drafted the manuscript, and TMM performed the statistical analysis. All authors read and revised the manuscript drafts and approved the final manuscript.
